# Editorial: Epigenetics of polycystic ovary syndrome

**DOI:** 10.3389/fendo.2023.1284351

**Published:** 2023-09-11

**Authors:** Jim Parker, Michal Kunicki

**Affiliations:** ^1^ School of Medicine, University of Wollongong, Wollongong, NSW, Australia; ^2^ Department of Gynecological Endocrinology, Medical University of Warsaw, Warsaw, Poland

**Keywords:** polycystic ovary syndrome, epigenetics, genetics, metabolism, reproduction, lifestyle

## Introduction

Polycystic ovary syndrome is a common metabolic and reproductive condition that is increasingly being recognized as an evolutionary mismatch disorder that manifests after exposure to a range of adverse lifestyle and environmental exposures (1, 2). Many of the pathophysiological changes observed in PCOS (chronic inflammation, insulin resistance, and hyperandrogenism) are mediated by cellular signalling molecules and metabolic intermediates that activate transcription factors involved in modulating the expression of specific genes (3). Epigenetic processes (DNA methylation, histone modification, microRNA) modify the structure of chromatin and differentially regulate the expression of PCOS susceptibility genes in response to changes in the internal and external environment (4).

Epigenetic regulation of gene function therefore provides the molecular mechanism for linking gene expression to lifestyle, diet, environmental and pharmaceutical modulators. Many of the biochemical, metabolic, and endocrine features of PCOS are reversible following modification of nutritional, exercise, sleep, stress, and psychological factors, as recommended in the international guidelines (5). The initial biochemical, endocrine, and clinical manifestations of PCOS therefore represent functional changes in cell signalling and epigenetic regulatory processes, that can be reversed by lifestyle and pharmaceutical interventions (1–6).

## The main points of the individual contributions

This series of six papers evaluate a range of epigenetic mechanisms related to metabolic, reproductive, and lifestyle-related factors in women with PCOS.

Azumah et al. examined the expression patterns of PCOS candidate genes by measuring steady-state mRNA levels in gonadal, metabolic, and cerebral tissue. PCOS candidate genes were grouped according to their cellular functions (cellular, enzymatic, cell surface receptors, extracellular matrix regulation, metabolism, and reproduction), during fetal development and postnatally until adulthood. They found that the genes were dynamically expressed in fetal tissues at different time points prenatally and/or postnatally. They concluded that the fetal origin of a predisposition to PCOS, could arise from the effects of PCOS candidate genes during the development of multiple organs, and recommended further investigation using transcriptome-wide and Mendelian randomisation studies.

Tong et al. reviewed the role of the PI3K signalling pathway in insulin resistance, autophagy, and apoptosis in women with PCOS. The development of insulin resistance in women with PCOS can be due to several mechanisms, including impaired PI3K signalling. This review provides data that suggest a role for impaired PI3K signalling in the hypothalamus, pancreas, endometrium, and ovarian granulosa cells, in animal models and women with PCOS. The authors also review several therapeutic treatment modalities that may improve insulin sensitivity via their action on the PI3K pathway and the related autophagy/apoptosis signalling network.

Di et al. performed a metabolic study on subcutaneous adipose tissue samples from women with PCOS compared to matched controls. Their untargeted analysis showed that 107 metabolites were enriched in women with PCOS. The main classes of differential metabolites were amino acids and peptides, with mass spectrometry showing low isoleucine levels. The pathway enrichment analysis showed that the differential metabolites were mainly enriched in inflammatory diseases and mitochondrial beta-oxidation, both of which have been implicated in the pathophysiology of PCOS. In addition, the authors showed that the addition of isoleucine to a DHEA-induced granulosa cell culture model, promoted cell proliferation and replenished mitochondrial function. The authors suggested that candidate amino acids may have diagnostic and therapeutic capacity in women with PCOS.

Han et al. explored the effect of time-restricted feeding (TRF) on metabolic and endocrine profiles in a PCOS mouse model. They compared mice fed a high-fat diet ad libitum with a time-restricted protocol. The data indicated that TRF significantly improved glycolipid metabolism, hyperandrogenemia, the menstrual cycle, and ovarian morphology, in an obese PCOS mouse model. The TRF group had significantly reduced body weight and decreased fat mass. The authors point out the potential limitations of using a mouse model and the lack of mechanistic data in their study. Han et al. recommend that this lifestyle intervention requires further investigation in women with PCOS.

Zhang et al. performed a bidirectional Mendelian randomization (MR) study to evaluate a possible association between 25-hydroxyvitamin D (25OHD) and PCOS. Single nuclear polymorphisms were identified from genome-wide association studies of European women with PCOS. Univariate MR analysis showed that genetically predicted PCOS was weakly inversely associated with deficiency of 25OHD. The weak causal effect disappeared on multivariate MR analysis after adjusting for the effect of obesity and insulin resistance. The authors concluded that the causal effect of PCOS on 25OHD may be mediated by the negative effect of obesity and insulin resistance on 25OHD. They identify several limitations in their study including small sample size, lack of data on clinical phenotypes, possible bias from gene pleiotropy, and participants from European ancestry.

Kong et al. performed a transcriptome-wide analysis of the NCBI Gene Expression Omnibus database of granulosa cells, to compare differential adenosine-to-inosine (A-to-I) RNA editing in women with PCOS to control samples. Functional enrichment analysis showed that 259 genes exhibited reduced differential A-to-I editing between PCOS and control granulosa cells. The differentially edited genes were mainly related to apoptosis and necroptosis pathways, both of which have been reported to be linked to PCOS. The authors concluded that post-transcription A-to-I editing may have an epigenetic regulatory role in the pathophysiology of PCOS. If confirmed, these findings could provide valuable insights into more targeted treatment options for PCOS.

## Synthesis and conclusions

This Research Topic has highlighted areas of current research on the epigenetics of PCOS. The contributing authors have presented findings related to important aspects of the developmental origins, metabolic, and lifestyle components of PCOS. This Research Topic also identifies a range of epigenetic mechanisms that may be involved in the development of PCOS. The authors have explored emerging epigenetic research methods and identified several areas that could be further investigated to help understand the role of epigenetics in the pathogenesis and pathophysiology of PCOS.

## Author contributions

JP: Writing – original draft. MK: Writing – review & editing.
